# Prognostic Significance of E-Cadherin Expression in Hepatocellular Carcinoma: A Meta-Analysis

**DOI:** 10.1371/journal.pone.0103952

**Published:** 2014-08-05

**Authors:** Jiang Chen, Jie Zhao, Rui Ma, Hui Lin, Xiao Liang, Xiujun Cai

**Affiliations:** 1 Department of General Surgery, Institute of Minimally Invasive Surgery, Sir Run Run Shaw Hospital, College of Medicine, Zhejiang University, Hangzhou, Zhejiang, China; 2 Department of Surgery, Zhejiang University Hospital, Zhejiang University, Hangzhou, Zhejiang, China; The University of Hong Kong, Hong Kong

## Abstract

**Backgrounds:**

Hepatocellular Carcinoma (HCC) is one of the most common malignancy of liver and HCC-related morbidity and mortality remains at high level. Researchers had investigated whether and how reduced E-cadherin expression impacted the prognosis of patients with HCC but the results reported by different teams remain inconclusive.

**Methods:**

A systematic literature search was performed in all available databases to retrieve eligible studies and identify all relevant data, which could be used to evaluate the correlation between reduced E-cadherin expression and clinicopathological features and prognosis for HCC patients. A fixed or random effects model was used in this meta-analysis to calculate the pooled odds ratios (OR) and weighted mean differences (WMD) with 95% confidence intervals (CI).

**Results:**

Total 2439 patients in thirty studies matched the selection criteria. Aggregation of the data suggested that reduced E-cadherin expression in HCC patients correlated with poor 1-, 3- and 5-year overall survival. The combined ORs were 0.50 (n = 13 studies, 95% CI: 0.37–0.67, Z = 4.49, P<0.00001), 0.39 (n = 13 studies, 95% CI: 0.28–0.56, Z = 5.12, P<0.00001), 0.40 (n = 11 studies, 95% CI: 0.25–0.64, Z = 3.82, P = 0.0001), respectively. Additionally, the pooled analysis denoted that reduced E-cadherin expression negatively impacts recurrence-free survival (RSF) with no significant heterogeneity. The pooled ORs for 1-, 3- and 5- year RSF affected by down-regulated E-cadherin were 0.73 (n = 6 studies, 95% CI: 0.54–1.00, Z = 1.95, P = 0.05), 0.70 (n = 6 studies, 95% CI: 0.52–0.95, Z = 2.32, P = 0.02), 0.66 (n = 5 studies, 95% CI: 0.48–0.90, Z = 2.64, P = 0.008). And what’s more, reduced E-cadherin expression tended to be significantly associated with metastasis (OR = 0.31, 95% CI: 0.16–0.60, Z = 3.50, P = 0.0005), vascular invasion (OR = 0.76, 95% CI: 0.59–0.98, Z = 2.14, P = 0.03), advanced differentiation grade (OR = 0.31, 95% CI: 0.21–0.45, Z = 6.04, P<0.00001) and advanced TMN stage (T3/T4 versus T1/T2) (OR = 0.61,95% CI:0.38–0.98, Z = 2.05, P = 0.04).

**Conclusions:**

Reduced E-cadherin expression indicates a poor prognosis for patients with HCC, and it may have predictive potential for prognosis of HCC patients.

## Introduction

Hepatocellular carcinoma (HCC) is not only the seventh most frequent human malignant tumors, but also the second highest cause of cancer-related death from poles to poles. It was estimated that HCC had caused about 746,000 deaths in 2012 [1]–[5]. Despite the considerable advancement in new-developed therapies, the overall mortality and morbidity for HCC are high and the prognosis of patients remains disappointed [6]. On the one hand, it might be due to that the time of diagnosing HCC is always at the advanced stage; on the other hand, clinicopathological features of HCC, such as differentiation, tumor grade/stage, lymph node status, depth of tumor invasion, and metastasis all influence the prognosis of patients with HCC. Consequently, new biomarkers that could be used effectively to anticipate the prognosis of patients with HCC are in urgent need [7]–[10].

Nowadays, the role of cell adhesion molecules, such as cadherin, catenin, selectin, integrin, whose expression levels change dynamically in tumor and have much association with tumor invasion and metastasis, has attached more and more attention [11]–[14]. These molecules could serve as potential marker predicting the prognostic significance for patients with HCC.

E-cadherin is the major member of cell adhesion molecule family expressed by epithelial cells [15]. It is a transmembrane calcium-dependent cell adhesion protein with a molecular weight of 120-KD. E-cadherin regulates cell differentiation and maintains cell structure. Detected by immunohistochemistry, reduced E-cadherin expression has been observed in a wide variety of tumors, characterized by decreased epithelial cell adhesion and increased motility and invasiveness of tumor cells [16]–[21]. Vast work has been done to examine the correlation of reduced E-cadherin expression with prognostic significance for patients with HCC but no concensus was achieved to date [22], [23]. Consequently, basing on retrospective cohort studies, we carried out this meta-analysis to systematically and comprehensively investigate whether and how the reduced E-cadherin expression impacted prognosis of HCCs.

## Methods

### Study Selection

The Pubmed, Elsevier, Embase, Cochrane Library and Web of Science databases were searched systematically for all articles published between 1990 and April 3, 2014 using the following terms: “E-cadherin”, “Cadherins”, “CDH1”, “cadherin”, “HCC”, “hepatocellular carcinoma”, “hepatic tumor”, “hepatic cancer”, “liver cancer”, “liver tumor” and “liver neoplasms” with all possible combinations. Using these parameters, we filtered out all the eligible articles and looked through their reference lists for additional available studies. The multifarious but crucial task to conduct a systematic literature search was undertaken independently by two reviewers (JC and JZ).

### Criteria for Inclusion and Exclusion

To make this meta-analysis meet the high standards, studies had to fulfill the following criteria: (1) patients with distinctive hepatocellular carcinoma diagnosis by pathology but without restriction on age or ethnicity; (2) reduced E-cadherin expression was measured by immunohistological chemistry (IHC) or other methods in primary HCC tissues; (3) clinical trials or reports on E-cadherin expression study in HCC were published in English; (4) valid data were provided directly or could be calculated indirectly; (5) the study with the highest quality assessment was enrolled when trials on similar objects were reported many times.

Abstracts, editorials, letters and expert opinions, reviews without original data, case reports and studies lack of control groups were excluded. Studies and data were also excluded if: (1) articles about animals or cell lines; (2) the outcomes or parameters of patients were not clearly reported (e.g. omitting standard deviations (SDs) (3) conference records; (4) no related data required for necessary analysis; (5) overlapping articles.

### Data Extraction and Literature Quality Assessment

Independently, valid data were retrieved from eligible studies by two reviewers (JC and JZ) and relevant characteristics were listed as follows: (1) first author’s name; (2) publication date; (3) study population characteristics; (4) disease stage; (5) the methods used to evaluate E-cadherin levels; (6) corporations of antibody; (7) percentage of reduced E-cadherin expression (Table 1). All relevant text, tables and figures were reviewed for data extraction. Any divergence was ironed out by discussion with the third reviewer (RM) for final expectation of consensus. The quality of each included study was assessed by utilizing the Centre of Evidence-Based Medicine.

**Table 1:** Characteristics of studies included in the meta-analysis.

First author& year	No. ofpatients	Mean age	Gender(M/F)	Level ofevidence	Stage	Clinicopathologicalfeatures	Method	Clone number ofantibody (source)	Dilution	Reduced E-cad.expression (%)	Blind evaluation	Definitionstandard*	ProvidedOSdata
Zhang 2013	66	NR	60/6	3	I–III	D, T	IHC	(Santa Cruz Biotech, USA)	NR	38/66 (57.6)	Yes	NR	No
Xia 2013	406	NR	331/75	3	I–III	D, T	IHC	NR	NR	156/406 (38.4)	Yes	NR	Yes
Pan 2013	70	53 (21–74)	60/10	3	I–IV	D, T, M	IHC	(Zhongshan Golden BridgeBiotechnology, China)	NR	45/70 (64.3)	Yes	CS	No
Liu 2013	113	NR	NR	3	I–IV	D	IHC	(BD Biosciences, USA)	1∶800	24/113 (21.2)	Yes	CS	No
Hashiguchi 2013	108	65.3	85/23	3	I–IV	D, T, M	IHC	(DAKO, Japan)	1∶100	44/108 (40.7)	Yes	NR	Yes
Ding 2013	42	57.1±12.1 (33–82)	37/5	3	NR	D, T	IHC	(Takara, Japan)	NR	32/42 (76.2)	Yes	NR	No
Zhang 2012	100	55.1 (28–77)	80/20	5	I–IV	D, T, M	IHC	(Santa Cruz Biotech, USA)	1∶100	52/100 (52)	Yes	<90%	Yes
Jiang 2012	125	51.43 (18–75)	115/10	5	I–III	T, M	IHC	(Santa Cruz, USA)	1∶200	71/124 (57.3)	Yes	CS	Yes
Xia 2012	50	NR	NR	3	I–IV	NR	IHC	(Cell Signaling, MA)	NR	26/50 (52)	Yes	CS	No
Zhao 2011	97	NR	82/15	3	NR	M	IHC	(Santa Cruz Biotech, USA)	1∶100	48/97 (49.5)	Yes	NR	Yes
Woo 2011	59	57 (35–80)	48/11	3	I–IVA	D, M,	IHC	(DECMA-1, Cambridge, UK)	1∶500	33/59 (55.9)	Yes	CS	Yes
Du 2009	43	49 (29–72)	36/7	3	NR	D	IHC	(Abgent Biotechnology, CA)	1∶50	31/43 (72.1)	Yes	CS	No
Korita 2008	125	63 (16–79)	88/37	3	NR	D	IHC	Novocastra Laboratories Ltd, UK	1∶50	34/125 (27.2)	Yes	CS	Yes
Zhai 2008	97	54 (34–72)	67/30	5	I–IV	D, LN, M,	IHC	(Santa Cruz Biotech, USA)	1∶200	57/97 (58.8)	Yes	<5%	Yes
Wu 2008	41	52.929 (36–73)	35/6	4	I–IV	D, T,	IHC	(Zymed Laboratories San Francisco, CA, USA).	NR	21/41 (51.2)	Yes	CS	Yes
Guo 2008	40	NR	32/8	3	I–IV	D, LN	IHC	(Santa Cruz Biotech, USA)	NR	18/40 (45)	Yes	<50%	Yes
Cho 2008	68	60±9	55/13	4	I–IV	D	IHC	(Santa Cruz Biotech, USA)	1∶100	30/68 (44.1)	Yes	<30%	Yes
Higashi 2007	55	64 (40–80)	46/9	5	NR	D, M	IHC	(Transduction Laboratories, Lexington, KY)	1∶1000	21/55 (38.2)	Yes	CS	Yes
Yamaoka 2006	17	4.17	13/4	4	I–IV	D, M	IHC	(Takara, Japan)	5–10µg/ml	1/17 (5.88)	Yes	0	No
Liu 2005	196	52 (14–72)	137/59	3	NR	D	IHC	(Santa Cruz Biotech, USA)	1:600	83/196 (42.3)	Yes	CS	No
Kwon 2005	72	49.9 (26–66)	57/15	3	NR	D	IHC	(San Francisco, CA, USA)	1∶40	18/64 (28.1)	Yes	<50%	No
Herath 2004	61	50.8 (23–77)	51/10	3	NR	NR	IHC	(Santa Cruz Biotech, USA)	1∶200	1/61 (1.6)	Yes	CS	Yes
Lee 2003	60	53.8	47/13	3	NR	NR	MSP	NR	NR	20/60 (33.3)	Yes	NR	Yes
Wei 2002	63	54.8 (16–78)	55/8	3	NR	D	IHC	(Transduction Labs)	NR	13/37 (35.1)	Yes	0	No
Satoshi 2002	51	63.5 (45–79)	33/18	4	NR	D	IHC	(Takara, Japan)	1∶200	16/51 (31.4)	Yes	NR	Yes
Asayama 2002	29	60 (37–73)	28/1	4	NR	D, M	IHC	(Transduction Laboratories, USA)	1∶1000	7/24 (29.2)	Yes	<90%	No
Matsumura 2001	30	NR	NR	3	I–IV	D	Western bloting	(Takara, Japan)	NR	10/21 (47.6)	Yes	NR	No
Endo 2000	107	60 (17–80)	87/20	4	NR	D	IHC	(Takara, Japan)	1∶100	39/107 (36.4)	Yes	CS	Yes
Ihara 1996	41	60.1 (42–77)	38/3	3	NR	D	IHC	(Takara, Japan)	1 ug/ml	23/66 (53.5)	Yes	CS	No
Kazuaki 1995	7	NR	NR	3	NR	D	IHC	(Takara, Japan)	1∶800	1/7 (14.3)	Yes	NR	No

CS: complex score combining intensity and percentage; IHC: immunohistochemistry; MSP: Methylation Specific PCR; D: histologic differentiation degree; LN: lymph node metastasis; T: depth of tumor invasion; M: metastasis; OS: overall survival; NR: not reported; E-cad: E-cadherin;

*The definition standard of reduced E-cadherin expression.

### Statistical Analysis

This meta-analysis was performed using the Review Manager (RevMan) software (version 5.2; Cochrane collaboration, http:ims.cochrane.org/revman/download) and STATA (version 12.0, Stata Corp. College Station, Texas) [24]. Odds ratios (OR), together with 95% confidence intervals (CI), was analyzed to estimate whether and how reduced E-cadherin expression impacts the prognosis of HCCs. Pooled values of ORs and 95% CIs, as the recommended summary statistics for meta-analysis, were calculated using either a fixed-effects or a random-effects model. Heterogeneity among the outcomes of enrolled studies in this meta-analysis was evaluated by using Chi-square based Q statistical test [25]. And *I* ^2^ statistic was calculated to quantify the total variation consistent with inter-study heterogeneity, ranging from 0% to 100% Heterogeneity was significant and unacceptable while *I* ^2^ statistic was greater than 50%. P<0.05 in Q statistical test was considered statistically significant. Choose fixed-effects models to calculate effect size estimates for those studies lack of heterogeneity with a P value for Q-test higher than 0.10. On the contrary, random-effects models were used when P≤0.10. The funnel plots was made by utilizing Egger’s test and Begg’s test to examine the risk of potential publication bias. Then, trim and fill analyses were used to evaluate the stability of our meta-analysis results if the plots were asymmetrical.

## Results

### Selection of trials

**Figure 1:**
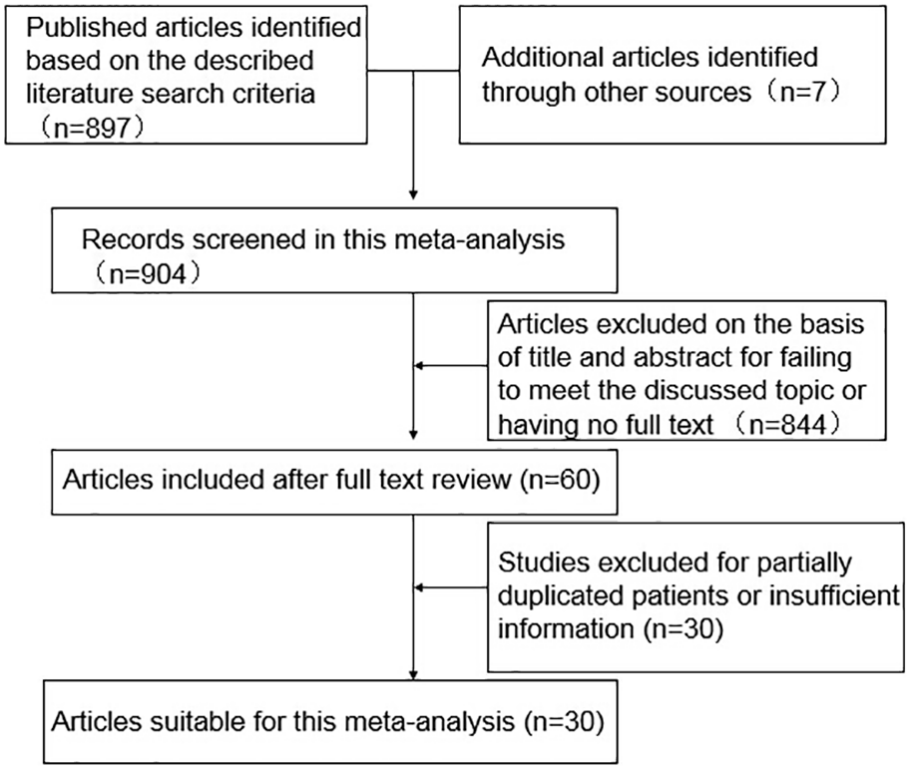
Flow chart of literature search strategies.

As shown in Fig. 1, 904 potentially eligible studies were screened out in the preliminary search. 844 articles were excluded for their improper titles and abstracts and 60 ones were captured after reviewing their full text for the relevance with the discussed topic. 30 studies was ultimately excluded due to a lack of clearly quantitative data on E-cadherin expression level in HCC. Thus, 30 studies, with more detailed and sufficient evaluation, met our entry criteria and were retrieved for further analysis. The flow diagram of study selection procedure was depicted in Fig. 1.

### Study Characteristics

The related clinical data of the enrolled 30 studies with a total of 2439 patients are depicted in Table 1. E-cadherin expression of 2415 tissue samples among the 2439 cases was determined successfully while that of the rest was not provided clearly. And 1013 tissue samples had reduced E-cadherin expression. The case size of each study varied from 7 to 406 patients. 20 studies [22], [23], [26]–[43] among these 30 ones scored 3 using the grading of the Centre of Evidence-Based Medicine (Oxford, UK; http:www.cebm.net/index.aspx?o=5653), 6 [44]–[49] scored 4, and the other 4 [50]–[53] scored 5. As shown in Table 2, all the 30 studies were evaluated blindly and the cases were grouped randomly according to the provided parameters without considering their age, gender, stage, pathological type and methods.

**Table 2:** Results of a meta-analysis comparing HCC with reduced E-cadherin and preserved E-cadherin.

Outcome of interest	No. of studies	Number of tissue samples	OR/WMD	95% CI	P value	I^2^ (%)
Overall Survival						
1 year	13 [22], [30], [34], [35], [38], [41], [42], [44], [49]–[53]	P.E-cad = 868, R.Ecad = 605	0.50	0.37–0.67	0.00001	43
3 year	13 [22], [30], [34], [35], [38], [41], [42], [44], [49]–[53]	P.E-cad = 868, R.Ecad = 605	0.39	0.28–0.56	0.00001	52
5 year	11 [22], [35], [38], [41], [42], [44], [49]–[53]	P.E-cad = 806, R.Ecad = 567	0.40	0.25–0. 64	0.0001	63
Recurrence-free Survival						
1 year	6 [22], [37], [39], [48], [50], [52]	P.E-cad = 425, R.Ecad = 337	0.73	0.54–1.00	0.05	0
3 year	6 [22], [37], [39], [48], [50], [52]	P.E-cad = 425, R.Ecad = 337	0.70	0.52–0.95	0.02	47
5 year	5 [22],[37],[39],[48],[52]	P.E-cad = 391, R.Ecad = 316	0.66	0.48–0.90	0.008	50
Differentiation grade	25 [22], [23], [26]–[29], [32]–[37], [40]–[51], [53]	P.E-cad = 1180, R.Ecad = 841	0.31	0.21–0.45	0.00001	56
Metastasis	10 [34], [38], [41], [43], [45], [47], [50]–[53]	P.E-cad = 368, R.Ecad = 364	0.31	0.16–0.60	0.0005	69
TMN stage	13 [22], [23], [28], [34], [37], [42], [43], [47]–[49], [51]–[53]	P.E-cad = 667, R.Ecad = 551	0.61	0.38–0.98	0.04	56
Liver cirrhosis	10 [22], [23], [31], [34], [40], [42], [43], [49], [52], [53]	P.E-cad = 605, R.Ecad = 453	0.92	0.69–1.24	0.58	19
Tumor encapsulation	7 [22], [23], [40], [42], [43], [50], [52]	P.E-cad = 482, R.Ecad = 384	0.84	0.62–1.14	0.25	0
Vascular invasion	12 [22], [23], [29], [37], [40]–[42], [45], [49]–[52]	P.E-cad = 655, R.Ecad = 515	0.76	0.59–0.98	0.03	16

P.E-cad: Preserved E-cadherin; R.E-cad: Reduced E-cadherin; *OR* odds ratio; *WMD* weighted mean difference; *CI*: confidence interval.

### Meta-Analysis of Overall Survival and Recurrence-free Survival

**Figure 2:**
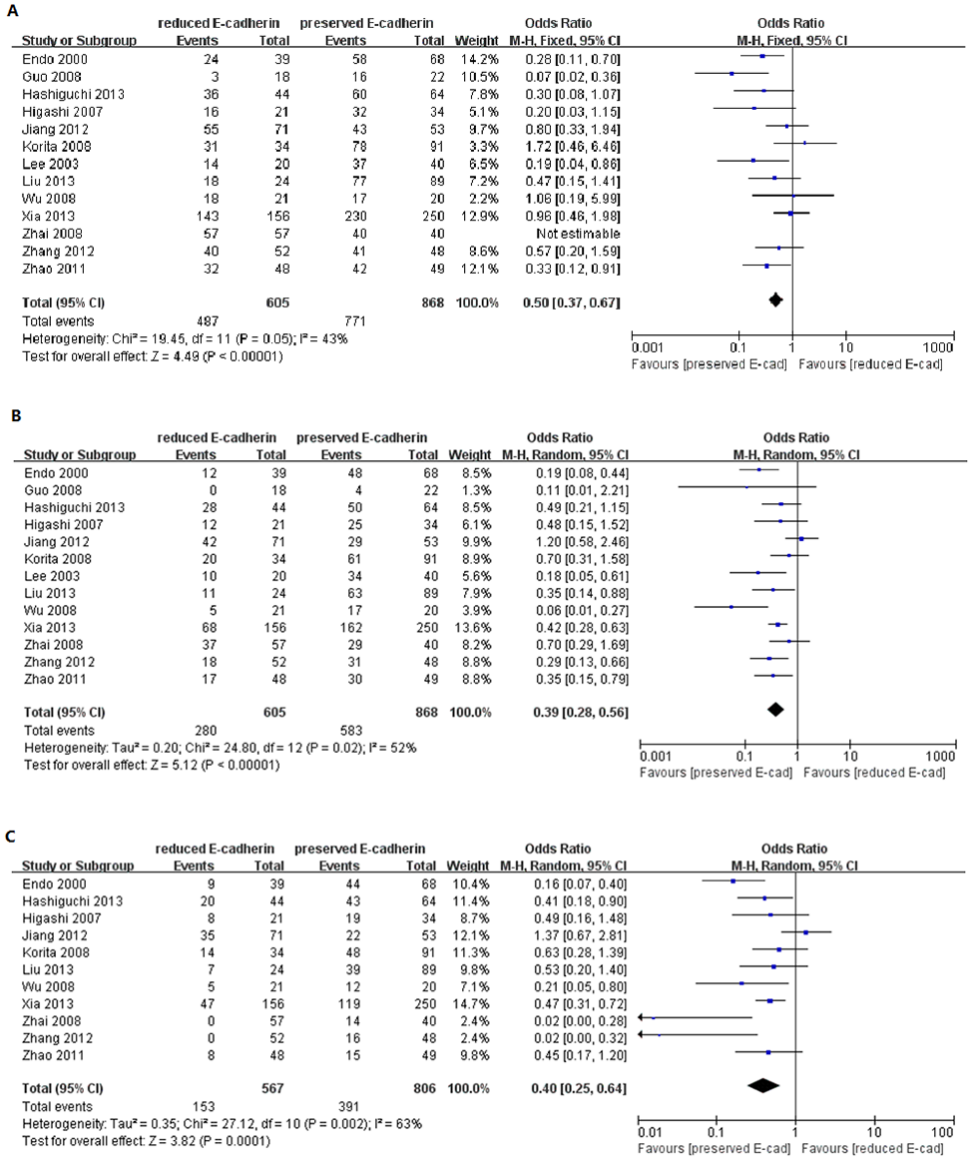
Forest plot displaying the results of the meta-analysis on 1-year (A), 3-year (B) and 5-year (C) overall survival.

**Figure 3:**
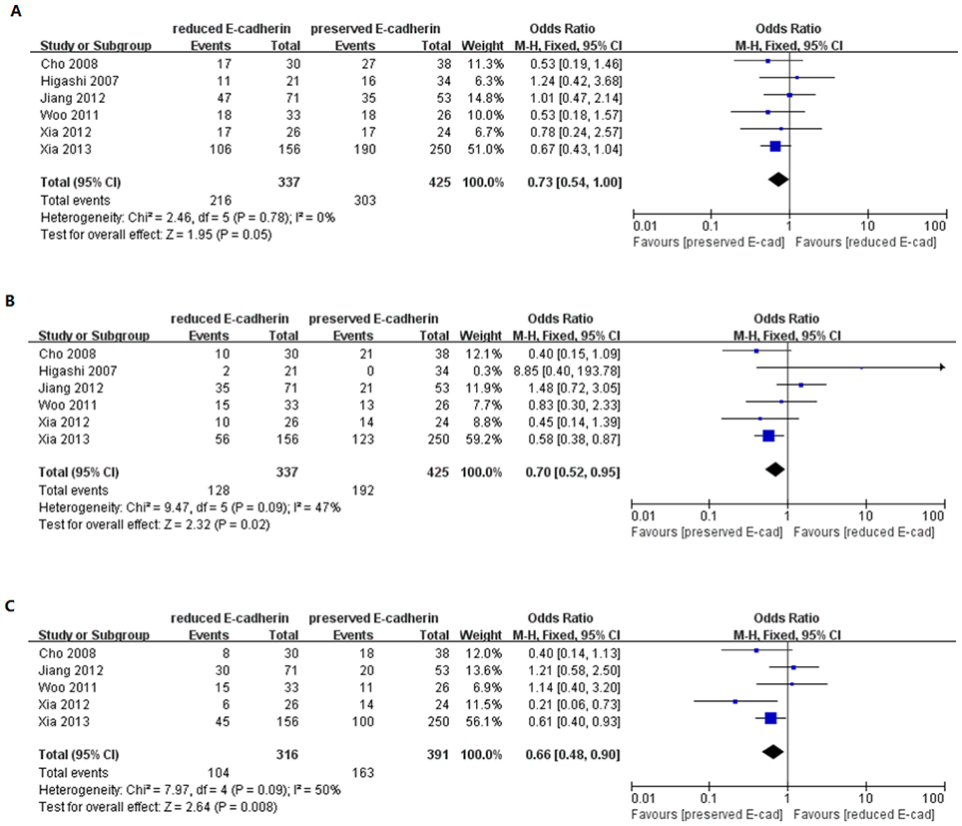
Forest plot displaying the results of the meta-analysis on 1-year (A), 3-year (B) and 5-year (C) recurrence-free survival.

As shown in Fig. 2 (A–C), based on 13 studies [22], [30], [34], [35], [38], [41], [42], [44], [49]–[53], we investigated whether and how E-cadherin expression impacted overall survival of patients with HCC by phasing three periods, one-year, three-year and five-year, respectively. The pooled analysis suggested that reduced E-cadherin expression correlated with lower overall survival for patients with HCC regardless of long or short term. The combined ORs were 0.50 (n = 13 studies, 95% CI: 0.37–0.67, Z = 4.49, P<0.00001), 0.39 (n = 13 studies, 95% CI: 0.28–0.56, Z = 5.12, P<0.00001), 0.40 (n = 11 studies, 95% CI: 0.25–0.64, Z = 3.82, P = 0.0001), respectively. Moreover, we also scrutinized the relationship between E-cadherin expression and recurrence-free survival for patients with HCC. Many studies provided the ORs and 95% CI for recurrence-free survival impacted by E-cadherin expression directly or indirectly [22], [37], [39], [48], [50], [52]. The analysis was performed by classifying these studies into three groups characterized by one-year, three-year or five-year period, as shown in Fig. 3 (A–C). The results showed that reduced E-cadherin expression was associated with poor recurrence-free survival with no significant heterogeneity (*I* ^2^ = 0%, 47% and 50%). The pooled ORs were 0.73 (n = 6 studies, 95% CI: 0.54–1.00, Z = 1.95, P = 0.05) for1-year RFS, 0.70 (n = 6 studies, 95% CI: 0.52–0.95, Z = 2.32, P = 0.002) for 3-year RFS, 0.66 (n = 5 studies, 95% CI: 0.48–0.90, Z = 2.64, P = 0.008) for 5-year RFS., Taken together, the above results suggested that reduced E-cadherin expression exerted a significantly adverse effect on the prognosis of patients with HCC.

### Meta-Analysis of Clinicopathology

In this meta-analysis, clinicopathologic features, such as differentiation grade, TMN stages, metastasis, vascular invasion, tumor encapsulation and liver cirrhosis, impacted by reduced or preserved E-cadherin expression was compared comprehensively on the basis of these 30 enrolled studies, in order to assess the association between E-cadherin expression and these clinicopathologic parameters. Some studies stated that lower E-cadherin levels unfavorably impacted clinicopathologic parameters [33], [43], [51] while the other studies found no significant effect [22], [29], [46], [47], [53]. Therefore, we carried out this meta-analysis with expectation of achievement of concensus about the correlation of E-cadherin expression and each clinicopathologic parameter.

**Figure 4:**
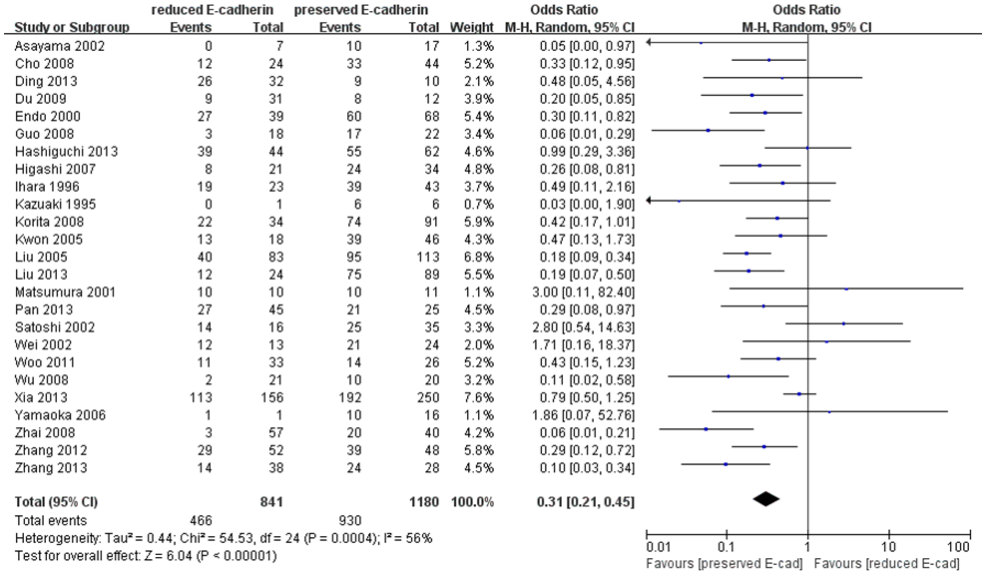
Forest plot displaying the results of the meta-analysis on differentiation grade.

**Figure 5:**
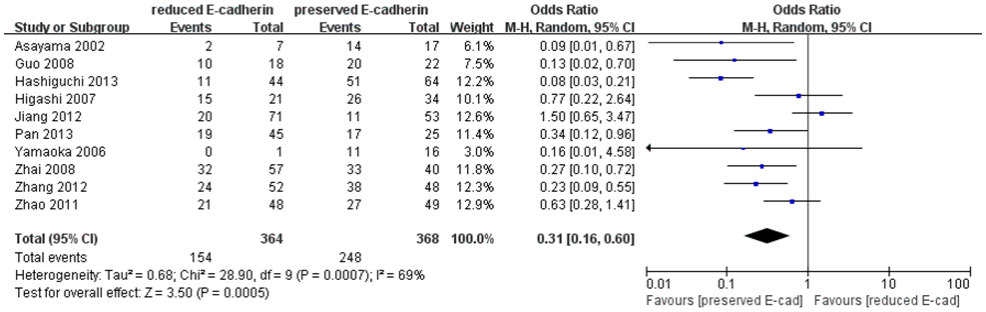
Forest plot displaying the results of the meta-analysis on metastasis.

**Figure 6:**
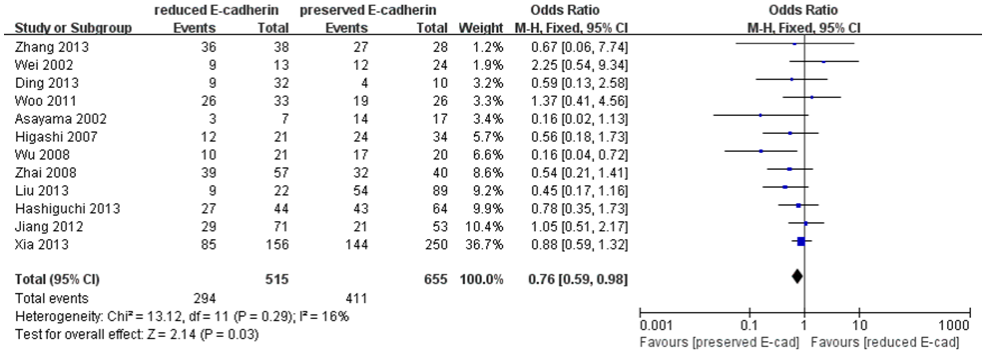
Forest plot displaying the results of the meta-analysis on vascular invasion.

**Figure 7:**
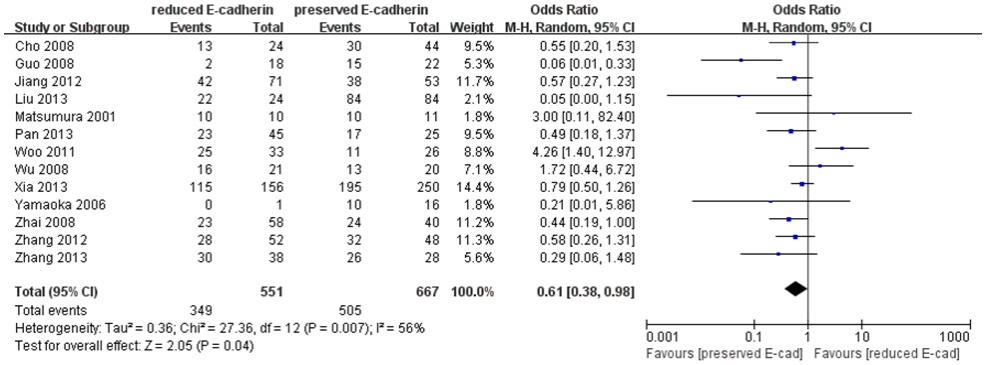
Forest plot displaying the results of the meta-analysis on TMN stage.

Twenty-five studies [22], [23], [26]–[29], [32]–[37], [40]–[51], [53] evaluated the impact of reduced E-cadherin expression on differentiation grade (III/IV versus I/II), as shown in Fig. 4. The combined OR was 0.31 (95% CI: 0.21–0.45, Z = 6.04, P<0.00001), indicating that down-regulated expression E-cadherin yielded advanced differentiation grade, correlated with poorer prognosis. Ten studies [34], [38], [41], [43], [45], [47], [50]–[53] assessing the effect of reduced E-cadherin expression on metastasis reported that reduced E-cadherin level was apt to cause metastasis (Fig. 5). The pooled OR was 0.31 (95% CI: 0.16–0.60, Z = 3.50, P = 0.0005) and statistical heterogeneity was significant (*I* ^2^ = 69%). As shown in Fig. 6, the pooled analysis based on other twelve studies [22], [23], [29], [37], [40]–[42], [45], [49]–[52] suggested that there was significant correlation between reduced E-cadherin expression and vascular invasion with no significant heterogeneity (*I* ^2^ = 16%). The pooled OR was 0.76 (95% CI: 0.59–0.98, Z = 2.14, P = 0.03). Moreover, thirteen studies [22], [23], [28], [34], [37], [42], [43], [47]–[49], [51]–[53] assessed the relationship of decreased E-cadherin level with TMN stages (T3/T4 versus T1/T2), as depicted in Fig. 7. The pooled OR was 0.61 (95% CI: 0.38–0.98, Z = 2.05, P = 0.04), indicating that there was comparatively significant correlation between decreased E-cadherin level with TMN stages (T3/T4 versus T1/T2). All the above results denoted that reduced E-cadherin expression would exert potential harm to clinicopathologic parameters. The down-regulated E-cadherin expression could serve as a prognostic predictor for patients with HCC.

**Figure 8:**
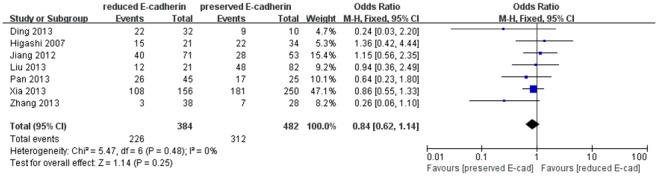
Forest plot displaying the results of the meta-analysis on tumor encapsulation.

**Figure 9:**
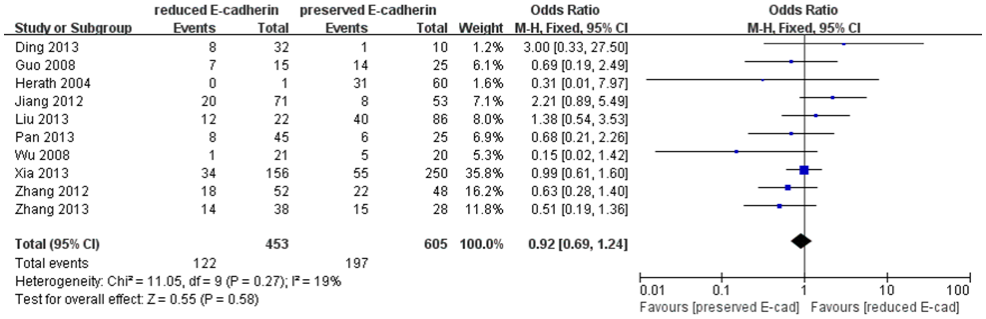
Forest plot displaying the results of the meta-analysis on liver cirrhosis.

Additionally, we also assessed the association of reduced E-cadherin expression with tumor encapsulation and liver cirrhosis on the basis of seven studies [22], [23], [40], [42], [43], [50], [52] and ten studies [22], [23], [31], [34], [40], [42], [43], [49], [52], [53], respectively. However, no significant association was found between reduced E-cadherin expression and poor tumor encapsulation (Fig. 8) and liver cirrhosis (Fig. 9) with no significant heterogeneity (0% and 19%). The combined ORs were 0.84 (95% CI: 0.62–1.14, Z = 1.14, P = 0.25), 0.92 (95% CI: 0.69–1.24, Z = 0.55, P = 0.58), respectively.

### Publication Bias

Begg’s test indicated that there was seemingly significant publication bias in OS and several other clinicopathologic parameters after assessing the funnel plot (Figure S1a–c, Figure S2a–c, Figure S3, Figure S4, Figure S5, Figure S6, Figure S7, Figure S8) for the studies included in our meta-analysis.

## Discussion

Meta-analytical technique is a useful tool in clinical researches and has been utilized more and more commonly. It can evaluate previous studies qualitatively and quantitatively, especially for those subjects still with controversial results, by integrating and comparing these results to estimate the outcome of interests. What’s worth mentioning, so far, tremendous work dedicated to investigating the relationship of E-cadherin levels and the prognosis of patients with HCC has been done with achieving no concensus. Therefore, we took the first effort to conduct a systematical and comprehensive meta-analysis to assess the relationship between them two. And it would also obviously provide useful information for clinical decision-making and effective targets for clinical therapies to treat HCCs.

Despite new therapies of HCC arising continually, the prognosis remains not very optimistic recently. That’s why many researchers have been dedicated to finding out predictors of prognosis. As is known to us all, many prognostic markers, such as surviving and MMP9, have been well studied. These markers could influence tumor metastasis and recurrence, the main two causes leading to poor prognosis. But unfortunately, all these markers alone could not predict the prognosis of patients with HCC reliably and exactly. So more iconic markers are needed as supplementary.

A newly-developed program, namely epithelial-mesenchymal transition (EMT) has been evidenced to participate in promoting progression and metastases of many epithelium-derived carcinoma including HCC [54]. During the process of EMT, epithelial cells actively downregulate cell–cell adhesion systems, lose polarity, and acquire a mesenchymal phenotype. This phenotype enables tumor cells to infiltrate surrounding tissues, and thus license these cells to metastasize in distant sites [21]. What’s more, snail and twist1 are the core transcription inhibitory factors during the process of EMT. The two factors can directly lead to reduced E-cadherin expression which is gradually became a hot spot in the field of cancer research. Downregulated E-cadherin expression indicates worse prognosis in some cancer [21]. E-cadherin is the major member of cell adhesion molecule family expressed by epithelial cells. It plays very important role in cell adhesion and differentiation [55]. According to the latest literatures [41], [42], [53], reduced E-cadherin expression had an adverse effect on the prognosis of patients with HCC and suggested that E-cadherin might be a factor to predict prognosis of patients with HCC.

We carried out this meta-analysis to examine whether and how E-cadherin level impacts the prognosis of patients with HCC. All available data are extracted from multiple databases including The Pubmed, Elsevier, Embase, Cochrane Library, and Web of Science. Low E-cadherin expression was observed in 41.95% of 2415 tissue samples included in our meta-analysis. Moreover, based on those extracted data, the association of reduced E-cadherin expression with OS, RFS, differentiation grade, metastasis, vascular invasion, TMN stage, tumor encapsulation and liver cirrhosis of HCCs was investigated. It was found that HCC with reduced E-cadherin expression became more aggressive and metastatic. Particularly, the results of this meta-analysis suggested that there was significant correlation between reduced E-cadherin expression and poor OS and RFS, indicating that reduced E-cadherin expression exerted a harmful effect on prognosis of patients with HCC. Moreover, lower E-cadherin level had significant correlation with metastasis, vascular invasion, advanced differentiation grade and TMN stages (T3/T4 versus T1/T2). But no significant association was found between lower E-cadherin level and poor tumor encapsulation and liver cirrhosis. All these results, taken together, denoted that reduced E-cadherin expression significantly correlated with poor prognosis of HCC.

However, some limitations need to be interpreted cautiously for further consideration in this meta-analysis. First, heterogeneity was inevitable among the groups due to impossibility to match patient characteristics in all studies. We used a random-effects model in order to eliminate variations across studies. Although it could not necessarily rule out the effect of heterogeneity among studies, the adverse influence will be weakened to some degree. Second, bias was unavoidable for clinical evidence because the relevant data were extracted from non-randomized controlled trials (NRCTs). The potential risks exist to weaken the results of large sample with better quality and strengthen the effect of the small sample with worse quality. Third, studies performed with positive results or significant outcomes will be apt to be published, suggesting a potential publication bias. Fourth, reports in other languages than English were excluded, so potential language bias may be present in our meta-analysis. Fifth, a significant heterogeneity might also be brought about in this meta-analysis by the difference of the antibodies used to test E-cadherin expression. Besides, other clinical characteristics of patients such as age, sex, different chemotherapies and radiotherapies in each study will obviously lead to bias. Further investigation should be given in determining whether these factors influence the results of the meta-analysis.

The prognostic significance of reduced or constant E-cadherin expression for HCCs was identified by comparing the depth of tumor invasion, lymph node metastasis, and clinical stage, cell differentiation, tumor grade and other clinicopathological features. Eventually, the data showed that reduced E-cadherin levels significantly associated with poor prognosis for patients with HCC. Thus, reduced E-cadherin expression may serve as a potential predictor for prognosis of patients with HCC.

## Supporting Information

Figure S1 **Funnel plot to assess publication bias. a.** Begg’s publication bias plot showed no publication bias for studies regarding *reduced* E-cadherin expression and 1-year overall survival (OS) in the meta-analysis. **b.** Begg’s publication bias plot showed the presence of publication bias for studies regarding *reduced* E-cadherin expression and 3-year OS in the meta-analysis. **c.** Begg’s publication bias plot showed the presence of publication bias for studies regarding *reduced* E-cadherin expression and 5-year OS in the meta-analysis.
(TIF)

Figure S2 **Funnel plot to assess publication bias. a.** Begg’s publication bias plot showed no publication bias for studies regarding *reduced* E-cadherin expression and 1-year reccurrence-free survival (RFS) in the meta-analysis. **b.** Begg’s publication bias plot showed no publication bias for studies regarding *reduced* E-cadherin expression and 3-year RFS in the meta-analysis. **c.** Begg’s publication bias plot showed no publication bias for studies regarding *reduced* E-cadherin expression and 5-year RFS in the meta-analysis.
(TIF)

Figure S3 **Funnel plot to assess publication bias.** Begg’s publication bias plot showed the presence of publication bias for studies regarding *reduced* E-cadherin expression and differentiation grade in the meta-analysis.
(TIF)

Figure S4 **Funnel plot to assess publication bias.** Begg’s publication bias plot showed the presence of publication bias for studies regarding *reduced* E-cadherin expression and metastasis in the meta-analysis.
(TIF)

Figure S5 **Funnel plot to assess publication bias.** Begg’s publication bias plot showed no publication bias for studies regarding *reduced* E-cadherin expression and vascular invasion in the meta-analysis.
(TIF)

Figure S6 **Funnel plot to assess publication bias.** Begg’s publication bias plot showed the presence of publication bias for studies regarding *reduced* E-cadherin expression and TMN stage ((III/IV versus I/II)) in the meta-analysis.
(TIF)

Figure S7 **Funnel plot to assess publication bias.** Begg’s publication bias plot showed no publication bias for studies regarding *reduced* E-cadherin expression and tumor encapsulation in the meta-analysis.
(TIF)

Figure S8 **Funnel plot to assess publication bias.** Begg’s publication bias plot showed no publication bias for studies regarding *reduced* E-cadherin expression and liver cirrhosis in the meta-analysis.
(TIF)

Checklist S1 (DOC)
