# Supplementary figures and images for: Prognostic Significance of E-Cadherin Expression in Hepatocellular Carcinoma: A Meta-Analysis
Source: PLoS One. 2014 Aug 5;9(8):e103952. doi: 10.1371/journal.pone.0103952 (PMC4122395; doi:10.1371/journal.pone.0103952)

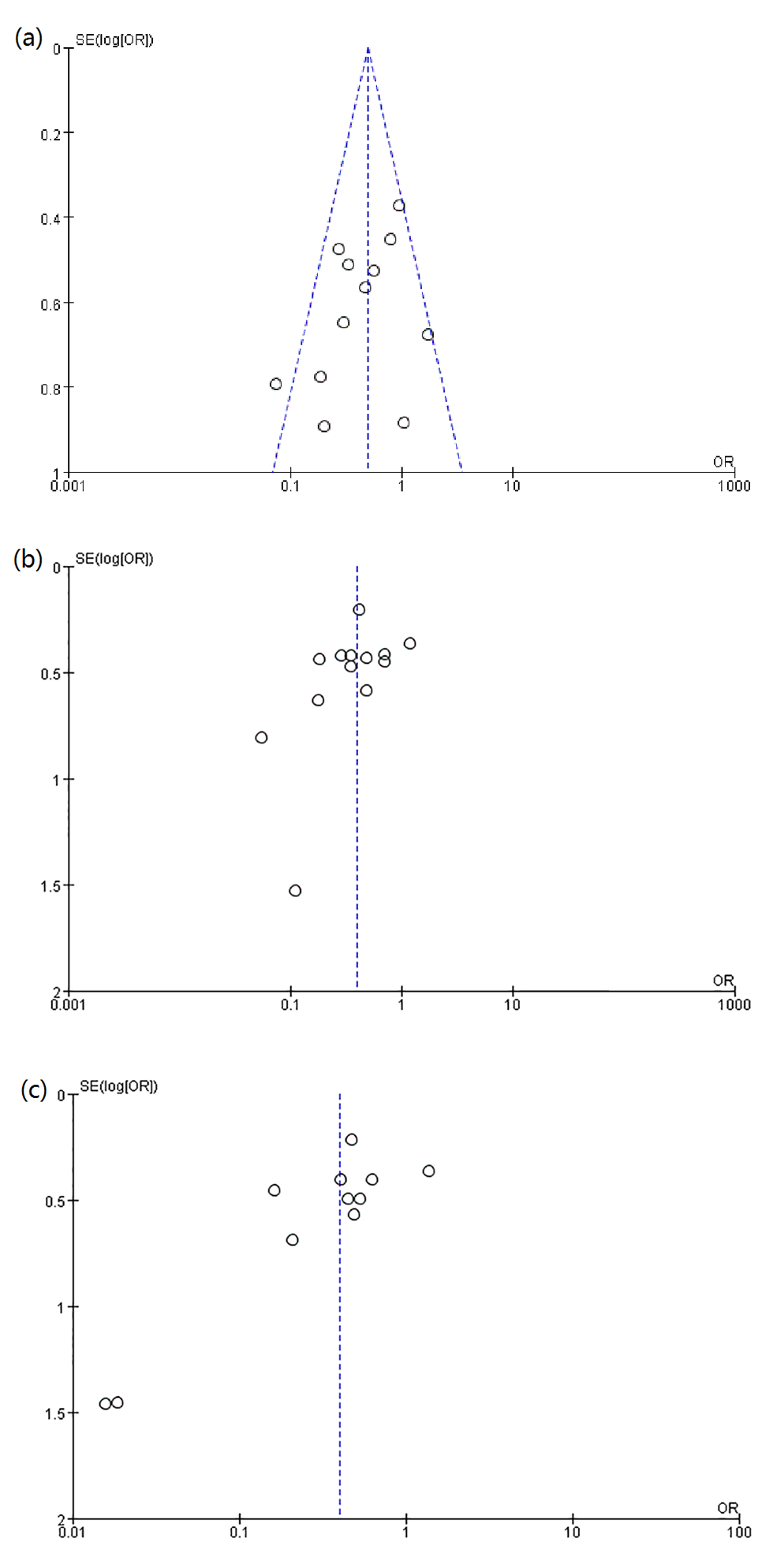

Supplement: Figure S1 — Funnel plot to assess publication bias. a. Begg’s publication bias plot showed no publication bias for studies regarding reduced E-cadherin expression and 1-year overall survival (OS) in the meta-analysis. b. Begg’s publication bias plot showed the presence of publication bias for studies regarding reduced E-cadherin expression and 3-year OS in the meta-analysis. c. Begg’s publication bias plot showed the presence of publication bias for studies regarding reduced E-cadherin expression and 5-year OS in the meta-analysis. (TIF) [file pone.0103952.s001.tif]

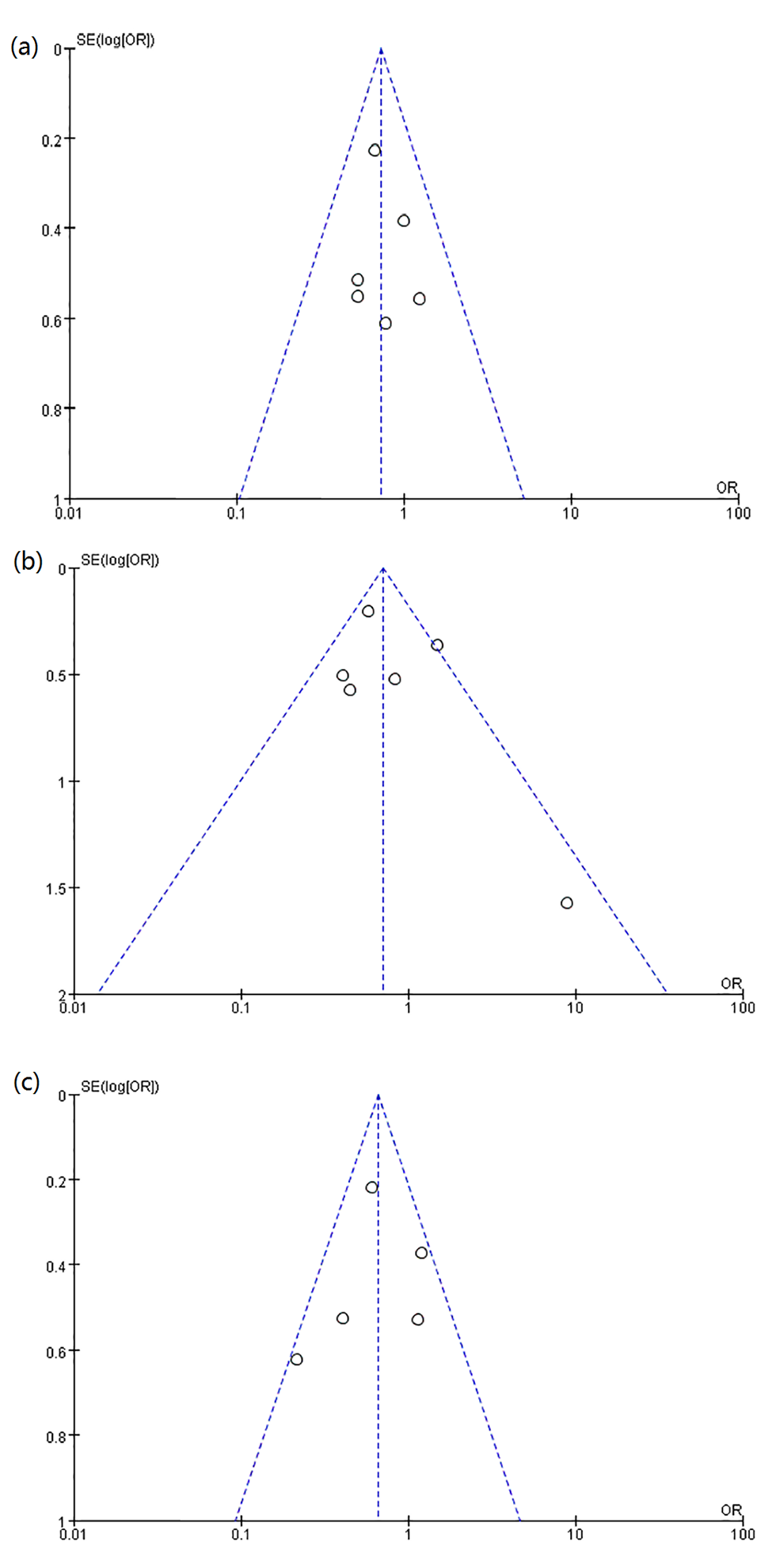

Supplement: Figure S2 — Funnel plot to assess publication bias. a. Begg’s publication bias plot showed no publication bias for studies regarding reduced E-cadherin expression and 1-year reccurrence-free survival (RFS) in the meta-analysis. b. Begg’s publication bias plot showed no publication bias for studies regarding reduced E-cadherin expression and 3-year RFS in the meta-analysis. c. Begg’s publication bias plot showed no publication bias for studies regarding reduced E-cadherin expression and 5-year RFS in the meta-analysis. (TIF) [file pone.0103952.s002.tif]

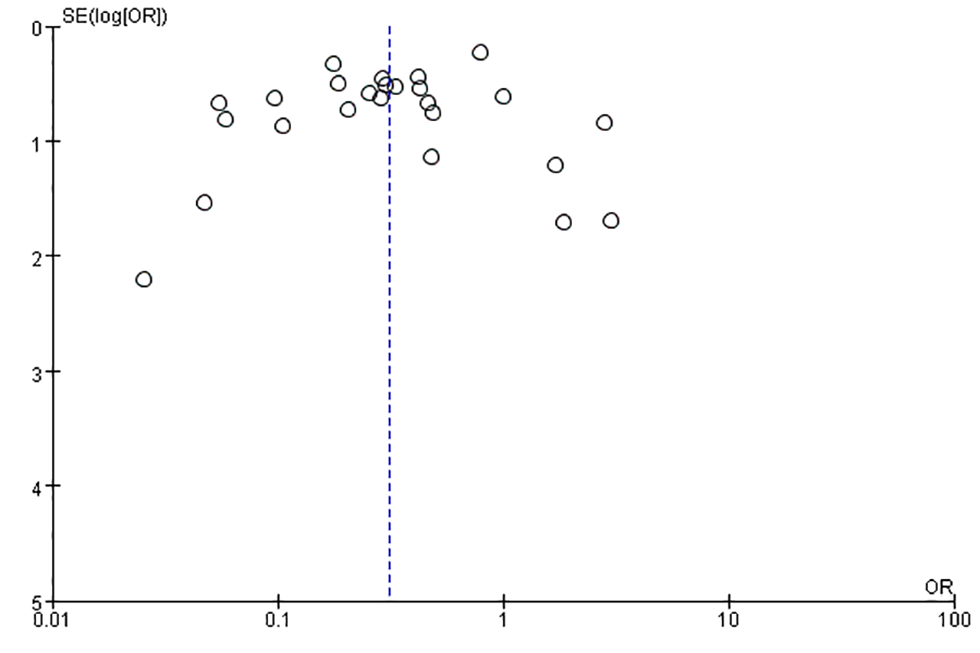

Supplement: Figure S3 — Funnel plot to assess publication bias. Begg’s publication bias plot showed the presence of publication bias for studies regarding reduced E-cadherin expression and differentiation grade in the meta-analysis. (TIF) [file pone.0103952.s003.tif]

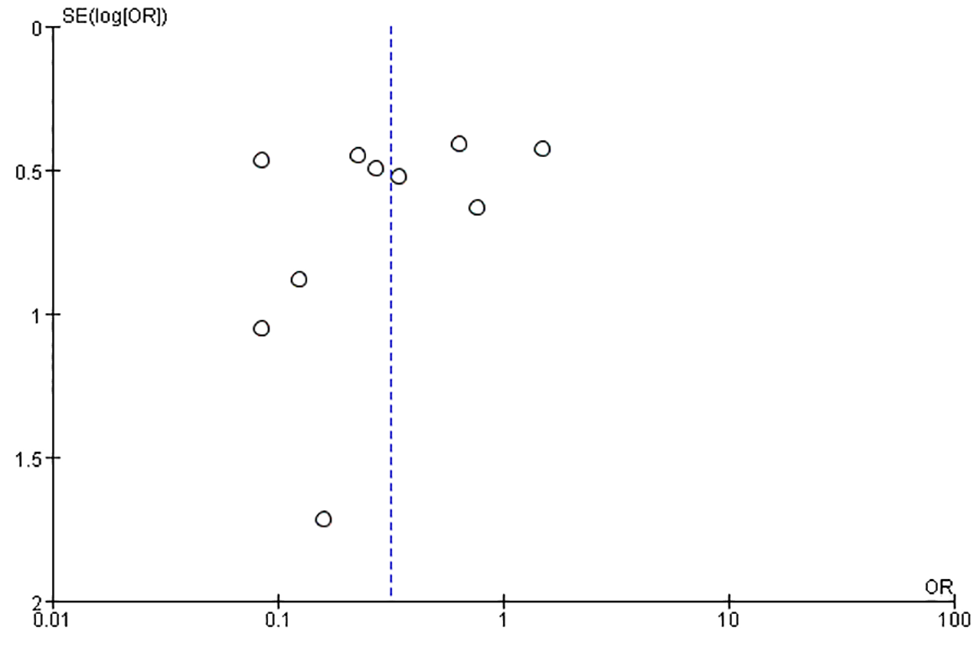

Supplement: Figure S4 — Funnel plot to assess publication bias. Begg’s publication bias plot showed the presence of publication bias for studies regarding reduced E-cadherin expression and metastasis in the meta-analysis. (TIF) [file pone.0103952.s004.tif]

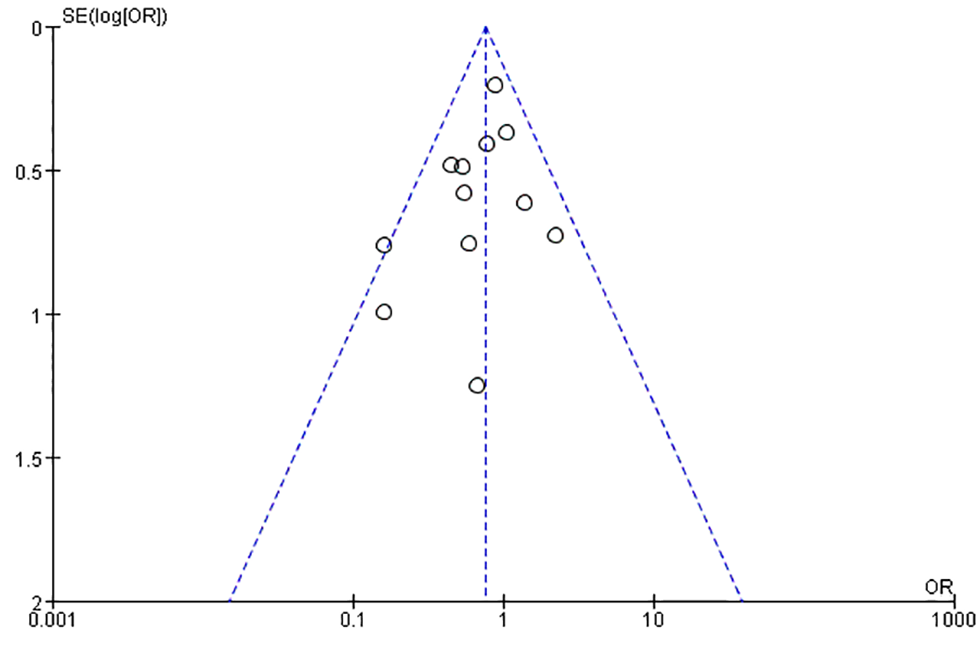

Supplement: Figure S5 — Funnel plot to assess publication bias. Begg’s publication bias plot showed no publication bias for studies regarding reduced E-cadherin expression and vascular invasion in the meta-analysis. (TIF) [file pone.0103952.s005.tif]

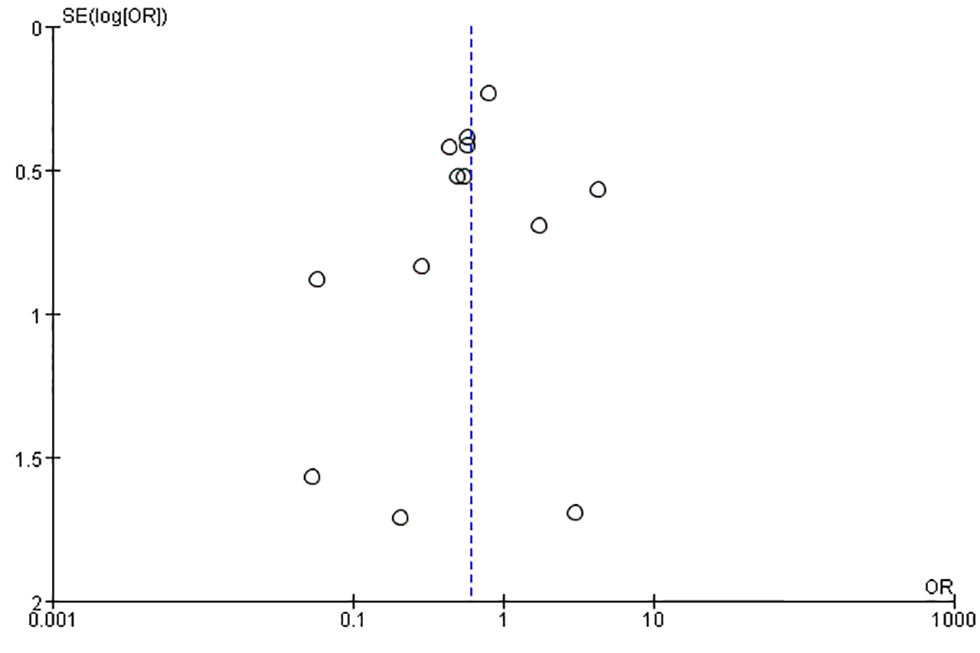

Supplement: Figure S6 — Funnel plot to assess publication bias. Begg’s publication bias plot showed the presence of publication bias for studies regarding reduced E-cadherin expression and TMN stage ((III/IV versus I/II)) in the meta-analysis. (TIF) [file pone.0103952.s006.tif]

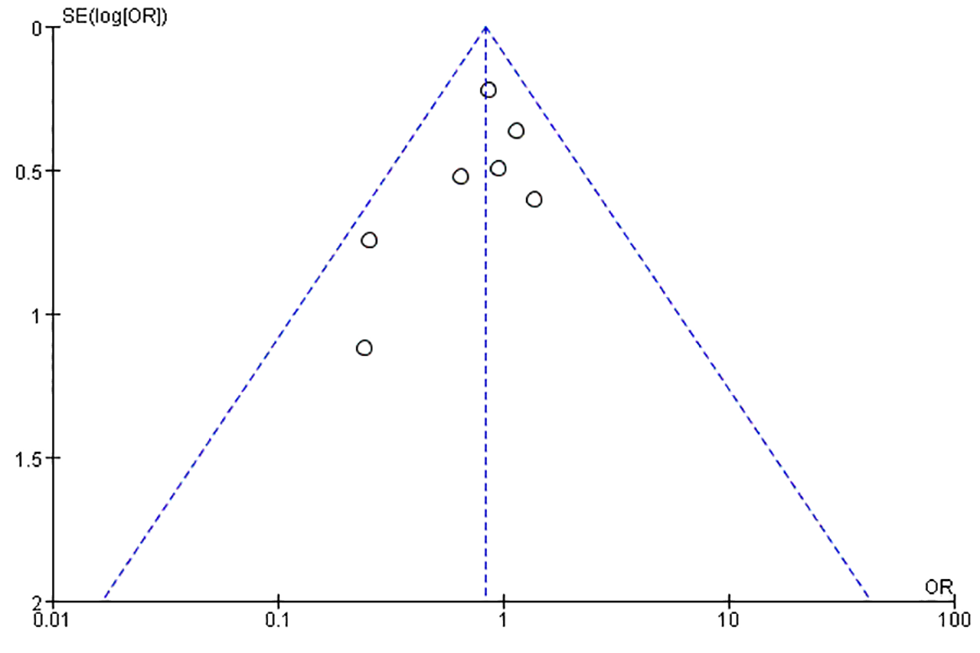

Supplement: Figure S7 — Funnel plot to assess publication bias. Begg’s publication bias plot showed no publication bias for studies regarding reduced E-cadherin expression and tumor encapsulation in the meta-analysis. (TIF) [file pone.0103952.s007.tif]

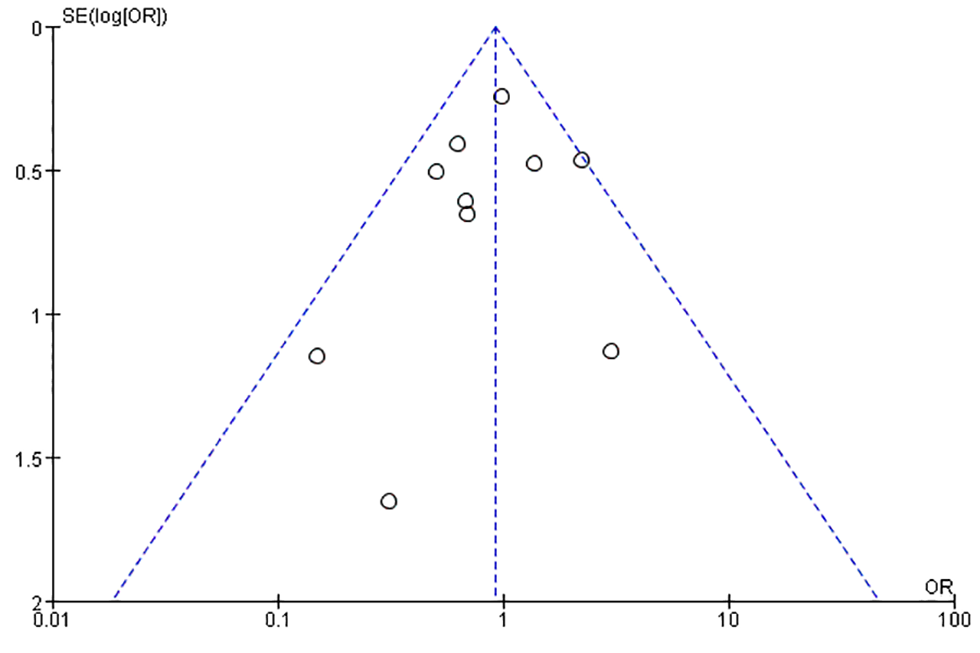

Supplement: Figure S8 — Funnel plot to assess publication bias. Begg’s publication bias plot showed no publication bias for studies regarding reduced E-cadherin expression and liver cirrhosis in the meta-analysis. (TIF) [file pone.0103952.s008.tif]
